# Antenatal Diagnosis and Management of Fetal Intestinal Volvulus: Case Series and Literature Review

**DOI:** 10.3390/jcm12144790

**Published:** 2023-07-20

**Authors:** Ramona Montironi, Valentina Tosto, Dayana Quintili, Daniele Crescenzi, Giovanna Irene Battistoni, Giovanni Cobellis, Stefano Raffaele Giannubilo, Andrea Ciavattini

**Affiliations:** 1 Clinical Sciences Department, Obstetrics and Gynecology Section, Università Politecnica delle Marche, Via Filippo Corridoni 11, 60123 Ancona, Italy; 2 Obstetrics and Gynecology Unit, IRCCS Istituto Giannina Gaslini, Via Gerolamo Gaslini 5, 16147 Genova, Italy; 3 Clinical Sciences Department, Pediatric Surgery Section, Università Politecnica delle Marche, Via Filippo Corridoni 11, 60123 Ancona, Italy

**Keywords:** fetal, intestinal volvulus, prenatal diagnosis, obstetric ultrasound

## Abstract

Fetal intestinal volvulus is a rare condition that can lead to hemorrhage, bowel necrosis, and urgent surgical treatment after birth. Thus, prompt diagnosis and treatment are essential to avoiding fetal or neonatal demise. Prenatal ultrasound is a keystone tool in the diagnostic course. However, sonographic findings tend to be non-specific, with limited understanding of the pathophysiology behind their atypical presentation. With a literature review and a case series, we aim to optimize the antenatal diagnosis and management of this rare but life-threatening condition. Six cases from our institution were retrospectively analyzed over 12 years. A literature review was conducted until December 2022. A total of 300 articles matched the keyword “Fetal volvulus”, and 52 studies were eligible for the review. Our 6 cases are added to the 107 cases reported in the literature of fetal intestinal volvulus with antenatal ultrasound assessment and without associated gastroschisis or omphalocele. Several prenatal symptoms and ultrasound markers, even if not specific, were more frequently reported. Different experiences of management were described regarding follow-up, the timing of delivery, the mode of delivery, and surgery outcomes. This paper highlights the importance of suspecting and assessing fetal volvulus at routine ultrasound scans, describing the most frequent antenatal presentations and management in order to improve fetal and neonatal outcomes.

## 1. Introduction

Volvulus occurs when bowel loops become twisted, and the twisting of the mesenteric vessels leads first to congestion and impaired venous return, then to hemorrhage and bowel necrosis, as a more severe degree of twisting inhibits arterial supply [1,2]. During fetal life, it is a rare condition, and its incidence is unknown. Predisposing factors are abdominal wall defects with gastrointestinal herniation (omphalocele and gastroschisis). It represents a surgical emergency, and delay in diagnosis or treatment can increase fetal/perinatal morbidity and mortality. However, early antenatal detection of this devastating condition is still challenging because it is rarely reported in the literature and because of a lack of specific fetal symptoms and ultrasonographic signs. It is, therefore, necessary to enhance antenatal diagnosis and management of this rare but life-threatening condition to improve fetal and neonatal outcomes. In this paper, we present a case series and a literature review on fetal intestinal volvulus with the aim of optimizing the diagnostic and management path by providing information about etiology, antenatal presentation (clinical and imaging) and management, postnatal presentation, and surgery outcomes. The objectives are: (1) to provide and summarize data on the antenatal characteristics of fetal intestinal volvulus, including fetal clinical presentation and ultrasound (US) signs; (2) to provide any information about volvulus etiology, gestational age (GA) at diagnosis, antenatal management, mode of delivery, postnatal presentation and treatment, surgery findings, and postoperative outcomes; and (3) to suggest how to manage fetal intestinal volvulus in light of the reported data.

## 2. Materials and Methods

In the first part of this paper, we analyzed 6 cases of fetal intestinal volvulus from our institution. In the second part, a literature review on fetal intestinal volvulus was conducted using Medline (PubMed) reports published until December 2022. According to the national legislation (Reg. EU no. 536/2014), ethical approval was not required because of the nature of this study (case series and systematic review). Standard written informed consent was obtained from all the subjects of the case series for the use of data, pictures, and video for teaching, research, and publishing purposes. 

### 2.1. Case Series

We retrospectively analyzed all cases of intestinal volvulus referred to our institution who had urgent surgical treatment after birth between January 2009 and February 2022. The sample was composed of cases of fetal intestinal volvulus without associated gastroschisis or omphalocele.

### 2.2. Literature Review

We performed an accurate literature search using PubMed/MEDLINE medical database. We used free-text search for keywords including only “fetal volvulus” until December 2022. Inclusion criteria were all human cases of fetal intestinal volvulus described in the scientific literature with prenatal US assessment and confirmed by pathology examination after neonatal surgery or autopsy. Exclusion criteria included all animal cases of intestinal volvulus, human cases of fetal volvulus without prenatal US assessment, cases without postnatal confirmation by pathology examination after surgery or autopsy, cases of fetal volvulus with associated gastroschisis or omphalocele (as prenatal volvulus occurs more frequently in these conditions), and cases of postnatal volvulus.

**Figure 1:**
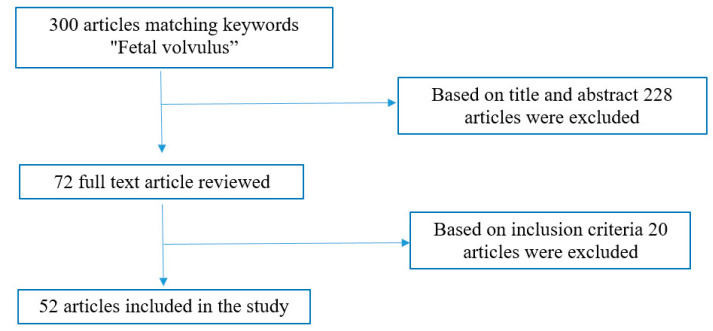
Outline of the literature review.

Titles and/or abstracts of the retrieved articles were screened to identify studies that potentially met the inclusion criteria. A total of 300 articles matched the keyword “fetal volvulus”. The full texts of these studies were retrieved and independently assessed for eligibility by the authors. Using the reported search modality, 228 articles were excluded based on title and abstract, 72 papers were identified independently and met inclusion criteria, and 20 articles were also excluded because they did not present inclusion criteria (among these, 12 were not available for consultation). In the end, a total of 52 studies met our inclusion criteria and were eligible for the review (Figure 1).

## 3. Results

### 3.1. Case Series Results

**Figure 2:**
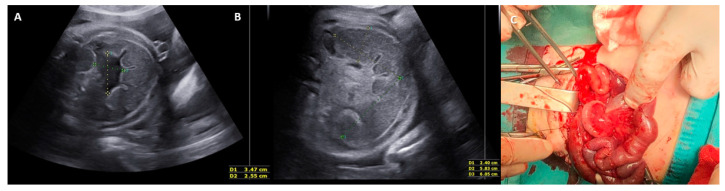
Case of fetal intestinal volvulus. (**A**,**B**) Fetal ultrasonography at 34 weeks of gestation: snail-like distended bowel loop with ascites; (**C**) intraoperative findings of the ileal volvulus with necrosis and distal atresia.

From January 2009 to February 2022, we found a total of six cases of fetal intestinal volvulus without associated major gastrointestinal defects. For each case, we collected its clinical and imaging presentation and its antenatal and postnatal management (Supplementary Table S1). Prenatal suspicion of fetal intestinal volvulus was questioned in two cases: one case of bowel dilatation (Figure 2) and one case of abdominal cyst (both noticed in the second trimester during a routine obstetrics scan). Except for one case where no prenatal data were available, in the other 3/6 cases, the diagnosis was made at birth due to the postnatal clinical presentation (bilious vomiting, tense and distended abdomen). Magnetic resonance imaging (MRI) was performed in only one case, helping in the differential diagnosis of the fetal abdominal cysts. Signs of fetal distress were found in 3/6 cases (Table 1). Regarding the etiology, two cases were due to intestinal duplication, two cases were related to ileal atresia, one case was idiopathic, and one case was due to malrotation (Table 2). Each case was managed individually with US follow-up (together with cardiotocography (CTG) after 28 GW) or immediate delivery in cases of worsening of the US features and/or signs of fetal distress (decreased fetal movements (FMs) and/or non-reassuring fetal heart rate (FHR)). All newborns required surgical treatment within 72 h of birth. With the exception of a volvulus of malrotated gut, where the Ladd’s procedure was performed, in all other cases, surgery consisted of resection and anastomosis of the midgut, with a temporary enterostomy when end-to-end anastomosis was not directly possible.

The postoperative outcome was uneventful in two cases. After surgery, the remaining cases developed intestinal obstructions (one case), short bowel syndrome (SBS) (two cases), and cholestasis (one case) (Table 3).

### 3.2. Literature Review Results

A total of 107 cases of fetal intestinal volvulus with antenatal US assessment and without abdominal wall defects (gastroschisis or omphalocele) had been reported in the literature up until December 2022. All data are derived from case reports and case series and are detailed in Supplementary Table S2. They include fetal volvulus clinical and imaging presentation, its antenatal and postnatal management, and postoperative outcomes.

#### 3.2.1. Clinical Presentation of Fetal Intestinal Volvulus

Prenatal suspicion or diagnosis was performed after US between 15 and 39 gestational weeks (GW), with a mean GA of 30 GW. The clinical context at diagnosis was during a routine obstetric scan or occurred through a sonographic examination performed because of a reduction in FMs and/or non-reassuring CTG, preterm labor, preterm premature rupture of membrane (PPROM), or suspicion of fetal abnormalities. Table 1 summarizes the clinical presentation of the fetal volvulus cases analyzed.

#### 3.2.2. Ultrasound Features of Fetal Intestinal Volvulus 

Except for 10 cases where no US abnormalities were found, antenatal suspicion and/or diagnosis were essentially made by US. MRI was performed in only nine cases, helping in the distinction of fluid peritoneal content, cystic mass content, and the detection of associated digestive anomalies. Hence, antenatal US features of fetal intestinal volvulus were detected in 97/107 cases and are summarized in Supplementary Table S3. Most of the cases (76/107) presented with the association of multiple US signs of fetal intestinal volvulus and only 21/107 cases with a single prenatal sign (bowel dilatation, whirlpool sign, hydramnios, ascites, stomach dilatation, coffee bean sign, meconium pseudocyst, or abdominal cystic mass) [3]. Overall, bowel dilatation was the most frequent US feature visualized in 78/107 cases, followed by ascites, found in 45/107 cases. The whirlpool sign was detected in 41/107 cases and disappeared during US follow-up in two of these cases, probably after bowel perforation.

#### 3.2.3. Etiology of Fetal Intestinal Volvulus

Table 2 reports the distribution of fetal volvulus etiology as defined by pathology examination after surgery or neonatal autopsy. Among the 107 cases, 39 were idiopathic, 25 were related to intestinal malrotation, 12 were related to cystic fibrosis (CF), 11 to minor gastrointestinal defects, 10 to small bowel atresia, 1 to the immaturity of intramural ganglion cells in the descending and sigmoid colon, 1 to meconium ileum, and 1 to a delayed return of the midgut to the abdomen beyond 13 GW. In seven cases, no definitive etiology was found, as it was not possible to establish if the associated minor gastrointestinal defect (e.g., intestinal atresia) was the cause or a consequence of the fetal intestinal volvulus.

#### 3.2.4. Antenatal Management of Fetal Intestinal Volvulus

Each case was managed individually with US follow-up (together with CTG after 28 GW) or immediate delivery in cases of worsening US features and/or signs of fetal distress (decreased FMs and/or non-reassuring FHR). Antenatal paracentesis was successfully performed in three cases in order to decompress the chest, prevent dystocia, and, in one case, discover the abdominal mass content. The termination of pregnancy occurred in five cases. There were two cases of intrauterine fetal death: one at 38 GW due to cardiovascular shock after midgut volvulus with signs of ascites after 15 GW, and the other at 39 GW due to ischemic hemorrhagic necrosis secondary to idiopathic midgut volvulus with no antenatal US manifestations.

#### 3.2.5. Postnatal Management of Fetal Intestinal Volvulus

There was only one case of postnatal spontaneous regression. In two cases, no data were available regarding the perinatal outcome. Two newborns died after postnatal reanimation: one died after spontaneous preterm delivery at 33 GW secondary to volvulus with complete necrosis of the ileum and subsequent intrauterine development of a pseudocyst and ascites, but only hydramnios was observed as prenatal US findings (this is probably due to the time the case dates back); the other equally died after spontaneous preterm delivery at 35 GW due to volvulus on malrotated intestine with necrosis of the twisted bowel and dissolution of twisted loops into a pseudocyst that was shown at prenatal US (10 cm cystic abdominal mass with septations and fluid-debris level, and hydramnios).

A total of 97 newborns required pediatric surgical management within 72 h of birth. Surgery consisted of resection and anastomosis of the midgut, with a temporary enterostomy when end-to-end anastomosis was not directly possible. The Ladd’s procedure was performed in cases of malrotation. Additional appendectomies were performed in very few cases. 

#### 3.2.6. Postoperative Outcome of Fetal Intestinal Volvulus

The postoperative outcome is summarized in Table 3. It was uneventful in 58/97 cases. A total of 29/97 cases developed postoperative complications: SBS (12 cases with large intestinal resection, 25–80 cm), sepsis (3 cases), peritonitis (2 cases), intestinal perforation (2 cases), cholestasis (2 cases), delayed continuity restoration (1 case), and intestinal obstructions (7 cases). Two cases developed respiratory complications due to CF. Death occurred in six cases. The six neonatal deaths after surgery involved:

1. One case of idiopathic midgut volvulus where the twisted bowel loop became a necrotized cyst mass of 7 cm in diameter. The baby died of multiorgan failure after spontaneous vaginal delivery and postnatal surgery at 30 GW. The fetus showed a 55 × 50 mm abdominal mass in the lower abdomen on US (with a thick wall and papillary projection) and signs of fetal distress (↑ peak of systolic velocity on the middle cerebral artery (PSV-MCA), preterm labor, and sinus rhythm).
2. One case of idiopathic midgut volvulus born after a cesarean section performed at 35 GW for non-reassuring FHR, despite the fetus showing severe signs suggestive of intestinal volvulus on US (whirlpool sign, fluid-meconium levels, bowel dilatation, meconium peritonitis) and signs of fetal distress (↓ FMs) after 34 GW.
3. One case of total volvulus on malrotated gut with necrosis, born at 38 GW after a spontaneous vaginal delivery and with no antenatal US findings.
4. One case of midgut volvulus due to partial malrotation and obstruction of the Meckel diverticulum. The baby died on day 7 because of volume and electrolyte imbalance due to SBS. The baby was born at 28 GW after a cesarean section performed for worsening of US features (hydramnios, multiple cystic spaces in the abdomen, ascites, ↑ abdominal circumference > 95° percentile) and after PPROM at 28 GW.
5. One case of idiopathic midgut volvulus died on day 10 after cardiac arrest. The baby was affected by Joubert syndrome and was born at 33 GW after a cesarean section performed for suspicion of fetal volvulus (dilated and hyperechoic bowel, gastric dilatation, mild ascites, and reduction in FMs).
6. One case of midgut volvulus with necrosis and perforation, intestinal atresia, and duplication. The baby died on day 36 of intraventricular hemorrhage and sepsis. The baby was born at 35 GW after a spontaneous vaginal delivery. A US follow-up was carried out from GW 34 despite the fetus showing signs of distress (preterm labor) and worsening of US findings of the volvulus, such as severe hydramnios, whirlpool sign (with no flow signal and subsequent disappearance), ↑ bowel dilatation, and thick ascites of mixed echogenicity.

## 4. Discussion

Midgut volvulus can occur during both the prenatal and postnatal periods. After birth, it usually occurs due to the malrotation of the bowel. However, during fetal life, it is a rare condition that more often occurs without malrotation [2,4] and may not have the usual signs of a volvulus with a malrotated gut [5]. Thus, the prenatal diagnosis of intestinal volvulus demands special consideration. Etiologies other than malrotation and congenital anomalies (omphalocele, gastroschisis, intestinal atresia, or annular pancreas) include idiopathic volvulus, delayed return of the fetal midgut to the abdomen beyond 13 GW, fibrous entero-umbilical band, segmental or basal mesenteric defects, bowel duplication, defects of the smooth muscle of the bowel wall, and segmental idiopathic bowel dilatation with torsion of the dilated segment. Moreover, prenatal intestinal volvulus may be due to CF because the dehydrated, thickened meconium obstructs the intestinal lumen. In our paper, most of the cases were idiopathic (40/113 cases), due to malrotation (26/113), or related to CF (12/113 cases). 

Suspicion of prenatal intestinal volvulus arises primarily in US. The fetal scan may show direct/typical and/or indirect/atypical signs. Direct findings of midgut volvulus are whirlpool and coffee bean signs (short bowel dilatation where the inner walls of the closed loop thicken and close to each other, forming a hyperechoic zone similar to coffee beans) [6,7,8]. Indirect signs are cystic abdominal masses, dilated bowel loops, peritoneal calcifications, ascites, and polyhydramnios [3,9,10,11,12,13]. If typical signs are absent, the differential diagnosis includes duplication cysts, giant Meckel diverticula, omphalomesenteric cysts, segmental small-bowel dilatation, cystic mass lesions (ovarian, teratoma, lymphangioma, and mesenteric cyst), intestinal atresia, and meconium peritonitis. In our paper, 13/113 cases presented with no abnormalities on US, no US data were available in 1 case, 49/113 cases presented with non-specific US signs, and 50/113 cases showed typical US findings. This suggests that fetal intestinal volvulus should also be questioned when non-specific abdominal abnormalities are found on US. We have indeed found bowel dilatation and ascites to be the most frequent antenatal findings, particularly when they appear together. Moreover, we think that the whirlpool sign should be considered a transient sign that can disappear as the disease progresses. This is suggested by two cases in the review where the absence of an identifiable vascularization on the Doppler examination resulted in the disappearance of the whirlpool sign due to necrosis and bowel perforation [3]. 

Prenatal complications of midgut volvulus could be lethal for the fetus, and knowledge about them is useful to understand the atypical presentations of volvulus on US and their clinical implications.

As mentioned above, a frequent complication of midgut volvulus is intestinal ischemic necrosis with consequent bowel perforation. Perforation usually occurs proximal to the intestinal obstruction and can cause the extrusion of the meconium into the abdominal cavity, resulting in meconium peritonitis. Depending on the different peritoneal inflammatory reactions, three types of meconium peritonitis are described with different US patterns: The “fibroadhesive type” is caused by an intense fibroblastic reaction. It is the most frequent type and is characterized by the presence of punctiform hyperechogenic lesions around the peritoneal cavity due to the formation of fibrotic membranes on the perforated intestinal wall. The “cystic type” is usually secondary to perforation and is the consequence of local meconium accumulation and adhesion in the bowel and greater omentum, with the result of pseudocyst formation. On US imaging, the pseudocyst seems to be a sizable meconium-filled cyst that is lined by a thick membrane that has numerous calcium deposits and plaques. The “generalized type” presents with ascites because of meconium spread throughout the abdominal cavity. The cystic type should be considered in the differential diagnosis of fetal cystic abdominal masses, and in our paper, it was present in 9/113 cases. The generalized type was present in 46/113 cases. Congenital midgut volvulus may be complicated, in about 25% of cases, by intestinal atresia [13]. When the volvulus occurs in utero, the resultant ischemia can lead to atresia of the associated bowel. However, it is also possible that intestinal atresia develops first, and then the increased peristalsis in the distended intestine proximal to the atresia creates the volvulus. That is why it is not always possible to establish if the atresia is the cause or the consequence of fetal intestinal volvulus, especially when the volvulus occurs distally from the atresia. We found 17/113 cases of fetal volvulus with associated atresia, and in only 12 of these, bowel atresia was certainly the causative agent of the volvulus. Other complications due to bowel infarction and necrotic perforation are hemorrhagic ascites, fetal anemia, and, eventually, fetal death. We found only two cases in which hemorrhagic ascites required postnatal transfusion (one was the only fetus presented as hydropic, and the other showed bowel dilatation, AC > 95° percentile, ascites, and hydramnios). Moderate postnatal anemia was found in four cases with ascites, bowel dilatation, hydramnios, and increased PSV-MCA on antenatal US. Thus, fetal anemia should always be questioned in cases of increased PSV-MCA, especially in the presence of ascites, polyhydramnios, and bowel dilatation. Prenatal intestinal volvulus is also responsible for fetal distress because ischemic necrosis might activate the release of stress hormones that can lead to preterm labor and delivery, PPROM, decreased FMs, late deceleration, poor variability on CTG, and increased PSV-MCA [5]. Sanders et al. [14] (p. 137) believe that bilious material aspiration into the lungs may also contribute to fetal distress. In our paper, the most common signs of fetal distress were decreased maternal perception of FMs and non-reassuring FHR on CTG (36/113 cases). The latter could therefore be considered symptoms of a worsening volvulus. It is worth noting that the presence on US of fluid-meconial levels in dilated bowel loops should also be considered a sign of fetal distress as it indicates the absence of peristalsis.

The postoperative outcome was uneventful or showed postoperative complications when delivery was performed immediately at worsening US findings with signs of fetal distress or only at worsening US findings when GA was over 34 GW. On the other hand, most of the six neonatal deaths were cases in which delivery was delayed despite worsening US findings and signs of fetal distress or cases in which delivery was performed only for worsening US findings despite extreme prematurity.

In light of the reported data, the diagnosis of fetal intestinal volvulus does not necessarily require immediate intervention. Delivery should be promptly performed in cases of coexistence of signs of fetal distress and worsening US findings. 

The prognosis depends on birth weight, GA at birth, the length of the affected bowel, and associated anomalies [5]. 

The suggested management of suspected/confirmed cases of fetal intestinal volvulus includes referral to a tertiary center with a neonatal surgical department and a multidisciplinary team planning for antenatal, intrapartum, and neonatal care [15]. Serial fetal monitoring at 28 GW and sonographic follow-up after 2 weeks should be performed. If the scans show progressive stabilization of the bowel loop dilation and the CTG/FMs are normal, an observational approach could be established. If there is a worsening of the US findings (particularly the appearance of ascites, fluid-meconial levels, and/or sudden changes in dilation of the intestinal bowel) and decreased FMs/CTG abnormalities, urgent delivery is advised regardless of the GA [7,16,17]. If the GA is >34 weeks, the delivery can be immediately programmed only at the worsening of US findings [16]. Vaginal delivery is not contraindicated. Some authors support fetal paracentesis in very preterm infants to improve fetal viability by decompressing the chest [18] or in fetuses with large abdominal circumferences to prevent dystocia and fetal distress [19]. 

## 5. Conclusions

Fetal intestinal volvulus is a very rare condition that often occurs without typical signs of postnatal volvulus and requires urgent surgical treatment after birth. Additionally, direct US findings may change and disappear as the disease progresses. Thus, the recognition of atypical signs helps to improve antenatal diagnosis and neonatal survival through prompt delivery and postnatal surgical intervention. The diagnosis should be especially questioned when bowel dilatation and ascites appear together on antenatal US. Cystic fibrosis must always be suspected, even in the absence of typical signs. Antenatal management depends strictly on the GA, the severity of each case, and the association with signs of fetal distress. Particular attention should be paid to maternal perception of FMs and FHR on CTG, as their alterations are the most common signs of fetal suffering due to the worsening of this condition. In conclusion, prenatal ultrasound confirms its importance as an instrument for detecting fetal intestinal volvulus. However, this paper highlights not only the necessity of suspecting and assessing the fetal volvulus at routine scans, even in the absence of typical signs, but also the importance of fetal clinical evaluation in guiding antenatal obstetric care for this rare but life-threatening condition. 

## Figures and Tables

**Table 1:** Clinical presentation of fetal volvulus.

Clinical Presentation	No. (%)		
	Review	Case Series	Total
ND	39	3	42 (37.1)
↓ FMs and/or non-reassuring CTG	33	3	36 (31.8)
N	19	0	19 (16.8)
↑ PSV MCA	7	0	7 (6.19)
Preterm labor	6	1	7 (6.19)
PPROM	6	1	7 (6.19)
IUFD	2	0	2 (1.7)

IUFD, intrauterine fetal death; ND, no data available; N, none; FMs, fetal movements; CTG, cardiotocography; PSV, peak systolic velocity; PPROM, preterm premature rupture of membranes; MCA, middle cerebral artery.

**Table 2:** Fetal volvulus presumed etiology.

Presumed Etiology		No. (%)	
	Review	Case Series	Total
Idiopathic	39	1	40 (35.3)
Malrotation	25	1	26 (23.0)
CF	12	0	12 (10.6)
Minor GI defects (persistent entero-mesenteric umbilical connection, mesentery fusion, mesentery dysplasia, primitive mesenteric breach, microcolon, hypoplastic distal segment of small bowel, partial malrotation with obstruction of Meckel diverticulum, bowel duplication)	11	2	13 (11.5)
Small bowel atresia	10	2	12 (10.6)
Not defined	7	0	7 (6.19)
Immaturity of intramural ganglion cells in descending and sigmoid colon	1	0	1 (0.8)
Meconium Ileum	1	0	1 (0.8)
Delayed return of midgut to abdomen beyond 13 weeks of gestation	1	0	1(0.8)
Total	107	6	113 (100)

No., number of cases; CF, cystic fibrosis; GI, gastrointestinal.

**Table 3:** Fetal volvulus postoperative outcome.

Postoperative Outcome	No. (%)		
	Review	Case Series	Total
Uneventful	58	2	60 (58.2)
Postoperative complications	29	4	33 (32.0)
short bowel syndrome	12	2	14
sepsis	3	0	3
peritonitis	2	0	2
perforation	2	0	2
cholestasis	2	1	3
delayed intestinal continuity restoration	1	0	1
intestinal obstruction	7	1	8
Neonatal death (including one total volvulus and one with Joubert syndrome)	6	0	6 (5.8)
Respiratory complications due to CF	2	0	2 (1.9)
No data available	2	0	2 (1.9)
Total	97	6	103 (100)

No., number of cases; CF, cystic fibrosis; GI, gastrointestinal.

## Data Availability

Not applicable.

## Supplementary Materials

The following supporting information can be downloaded at: https://www.mdpi.com/article/10.3390/jcm12144790/s1, Table S1: Cases of fetal intestinal volvulus with antenatal ultrasound assessment (from January 2009 to February 2022); Table S2: Human cases of fetal intestinal volvulus with antenatal ultrasound assessment (Review of Literature 1945–2022); Table S3. Ultrasounds features of fetal intestinal volvulus (Review of Literature). References [20,21,22,23,24,25,26,27,28,29,30,31,32,33,34,35,36,37,38,39,40,41,42,43,44,45,46,47,48,49,50,51,52,53,54,55] are cited in the Supplementary Materials.
