# Supplementary material for: Antenatal Diagnosis and Management of Fetal Intestinal Volvulus: Case Series and Literature Review
Source: J Clin Med. 2023 Jul 20;12(14):4790. doi: 10.3390/jcm12144790 (PMC10381374; doi:10.3390/jcm12144790)
Supplement: Supplementary file 1 [file jcm-12-04790-s001.zip › Table S1.pdf]

Table S1. Cases of fetal intestinal volvulus with antenatal ultrasound assessment (from January 2009 to February 2022)

Authors (Y)	Sample size	N°	Study design	Fetal comorbidities	Fetal US findings	Fetal MRI findings	Fetal clinical presentation	GA at diagnosis (W)	Treatment	Mode of Delivery	GA at birth (W)	Postnatal presentation	Postnatal treatment	Postnatal surgery findings	Post-operative outcome	Volvulus etiology
**Our cases (January 2009-December 2022)**	6	1	Case series	T1DM	N	N	non-reassuring CTG	36	Induction for non-reassuring CTG	Emergency CS for non-reassuring CTG	36	Bilious vomiting, tense and distended abdomen.	Detorsion, resection, ileostomy	Midgut volvulus with necrosis, ascites	Resurgery: resection for necrosis. SBS.	Cystic intestinal duplication
		2		ND	ND	ND	ND	ND	ND	ND	ND	ND	Detorsion, resection, ileo-ileal anastomosis	Ileal volvulus with necrosis and ileal atresia	Cholestasis	Ileal atresia
		3		MD twin pregnancy; TTTS treated with laser therapy at 16 w and death of a twin	N	N	Preterm labor, ↓CTGv	33	Emergency CS for non-reassuring CTG	CS during labour	33	Tense and distended abdomen	Resection, jejunostomy, colon biopsies	Jejunoileal volvulus with necrosis, meconium peritonitis	Stenosis of anastomosis, reanastomosis and adhesiolysis	Idiopathic
		4		N	N	N	N	41	N	spontaneous VD	41	Inconsolable crying, tense and distended abdomen, bilious vomiting	Detorsion, Ladd's procedure	Midgut volvulus with malrotation. ascites	U	Malrotation
		5		Interatrial defect (ostium secundum)	**Abdominal cyst** from II trimester (4x3x4 cm)	Intestinal duplication	N	20	US FU	spontaneous VD	38	Bilious vomiting, tense and distended abdomen	Detorsion, resection, , ileo-ileal anastomosis, appendectomy	Ileal volvulus with ileal duplication	U	Cystic ileal duplication
		6		N	21w → **hyperechoic intestinal wall** 22w→ **bowel dilatation** 33→↑ **bowel dilatation (19 mm) with thick and hyperechoic intestinal wall** 34 w→ ↑**bowel dilatation (27 mm) with snail-like sign, ascites, thick and hyperechoic intestinal wall (meconium peritonitis)**	N	↓FM, ↓CTGv, PPROM a 34 w	20	US FU	VD (induction for PPROM with brown AF)	35	RDS, Tense and distended abdomen	Reanimation, CPAP, detorsion, resection, ileostomy. Anastomosis after 2 m	Ileal volvulus with necrosis, perforation, meconium peritonitis and distal atresia	SBS	Ileal atresia

GA, gestational age; W, weeks; Y, years; D, days; M, months; H, hours; FU, follow up; CS, cesarean section; VD, vaginal delivery; MRI, magnetic resonance imaging; US, ultrasound; ND, no data available; N, none; FM, fetal movements; CTGv, cardiotocography variability; RDS, respiratory distress syndrome; CTG, cardiotocography; NICU, neonatal intensive care unit; CPAP, continuous positive airway pressure; LPT, laparotomy; CF, cystic fibrosis; FHR, fetal heart rate; PSV, peak systolic velocity; MCA, middle cerebral artery; IUFD, intrauterine fetal death; AC, abdominal circumference; TOP, termination of pregnancy; TTTS, twin-to-twin transfusion syndrome; MD: monochorial diamniotic (pregnancy); NST, non-stress tests; IUGR, intrauterine grow restriction; PPROM, preterm premature rupture of membranes; U, uneventful; SBS, short bowel syndrome; MOF. multiorgan failure; CR, continuity restoration; AF, amniotic fluid; T1DM, Type 1 diabetes mellitus.
