# Supplementary material for: Antenatal Diagnosis and Management of Fetal Intestinal Volvulus: Case Series and Literature Review
Source: J Clin Med. 2023 Jul 20;12(14):4790. doi: 10.3390/jcm12144790 (PMC10381374; doi:10.3390/jcm12144790)
Supplement: Supplementary file 1 [file jcm-12-04790-s001.zip › Table S2.pdf]

Table S2. Human cases of fetal intestinal volvulus with antenatal ultrasound assessment (Review of Literature 1945-2022)

**Authors (Y)**	**Sample size**	**N°**	**Study design**	**Fetal comorbidities**	**Fetal US findings**	**Fetal MRI findings**	**Fetal clinical presentation**	**GA at diagnosis (W)**	**Treatment**	**Mode of Delivery**	**GA at birth (W)**	**Postnatal presentation**	**Postnatal treatment**	**Postnatal surgery findings**	**Post-operative outcome**	**Volvulus Etiology**
**Fukushima et al. 2018 [20]**	1	1	Case report	N	Acute **gastric dilatation** (no bowel dilatation)	N	↓ FM, ↓CTGv	33	Emergency CS	CS	33	RDS, tachycardia, tense distended abdomen, Cullen's sign	Endotracheal intubation, resection, ileostomy	distal ileum volvulus with necrosis (no malrotation, no atresia)	U	Idiopathic
**Best et al. 2018 [15]**	1	2	Case report	N	Initially moderately dilateted stomach, distend bowel loops and aperistalsis → **progressive bowel dilatation** (up to 18 mm) with wall oedema , **whirlpool and coffe-bean sign**	N	↓ FM, non-reassuring CTG with mild tachycardia	32	Daily US FU	Spontaneous VD	32	RDS, pneumoperitoneum on day 2	CPAP, resection, anastomosis	Ileal volvulus with necrosis and meconium peritonitis	U	Idiopathic
**Sciarrone et al. 2015 [16]**	8	3	Case series	N	**Hydramions, bowel dilatation**	N	ND	21	US FU	CS (↑ dilatatio, ↑hydramnios)	35	ND	multiple intestinal resections, ileostomy	Volvulus at 30 cm from Treitz, peritonitis, multiple ileal atresias, abdominal pseudocyst	U	Idiopathic / Ileal atresia
		4		N	**Bowel dilatation**	N	ND	24	US FU	Spontaneous VD	40	ND	ileo-ileal anastomosis	Volvulus with ileal atresia, at 80 cm from Treitz, Meckel diverticulum	U	Idiopathic / Ileal atresia
		5		Single umbilical artery	**Bowel dilatation, ascites**	N	ND	23	US FU	CS (↑ dilatation , ascites appeearance, no peristalsis)	30	ND	Ileal resection, ileo-ileal anastomosis, appendectomy	Volvulus with ileal atresia, at 50 cm from Treitz, Meconial peritonitis	Respiratory complications of CF	CF

		6		Dilatated cisterna magna, hyperechoigenic diaphragmatic spots	**Whirpool sign**, **gastric dilatation, bowel dilatation, AC >95 th**	N	ND	33	Immediate CS	CS	33	ND	Ileal resection, ileo-ileal anastomosis	Volvulus without malrotation	U	CF
		7		N	**Hydramnios, coffee bean sign, gastric dilatation, Bowel dilatation, AC >95 th**	N	ND	33	US FU	CS	34	ND	Ileal resection, ileostomy, appendectomy	Volvulus without malrotation at 40 cm from Treitz (3twist), meconial peritonitis	Respiratory complications of CF	CF
		8		N	**Coffee bean sign, bowel dilatation, pseudocyst**	N	ND	33	Immediate CS	CS	33	ND	Ileal resection, ileo-ileal anastomosis, appendectomy	Volvulus at 25 cm from Treitz (2 twist forming pseudocyst)	U	Idiopathic
		9		N	**Hydramnios, bowel dilatation**	N	ND	30	US FU	CS	32	ND	Ileal resection, ileo-ileal anastomosis	Volvulus without malrotation at 60 cm from Treitz (3twist), meconial peritonitis, bowel dilatation and perforation	Peritonitis and intestinal necrosis at 3 w	Idiopathic
		10		N	**bowel dilatation, ascites, AC >95 th**	N	ND	32	US FU	CS	32	ND	resection, ileo-ileal anastomosis,	Volvulus with ileal atresia	Intestinal obstruction at 1 m	Idiopathic /Ileal atresia
**Durand et al. 2008 [21]**	3	11	Case series	N	**Hydramnios, bowel dilatation, whirlpool sign, ascites**	N	non-reassuring FHR	34	Immediate CS	CS	34	Tense distended abdomen	Detorsion, resection, double-barrel stoma	Midgut volvulus and perforation	Sepsis and seizures	CF

		12		N	**Bowel dilatatin, whirlpool sign, ascites**	N	non-reassurin g FHR	39	Immediate CS	CS during labour	39	Tense distended abdomen	Detorsion, resection, double-barrel stoma	Midgut volvulus	Intestinal obstructon + second resection	CF
		13		N	**Bowel dilatation, whirlpool sign, ascites**	N	ND	34	Planned CS	CS	ND	tense distended abdomen	Detorsion, resection, double-barrel stoma	Midgut volvulus	Intestinal obstruction +second stoma	CF
**Kaba et al.2015 [4]**	1	14	Case report	N	55x50 mm **abdominal mass** in lower abdomen (with **thick wall and papillary projection),↑P SV MCA**	N	Preterm labor, sinus rhythm, ↑PSV MCA	30	Maternal hydratation, corticostero ids	Spontane ous VD	30	RDS, tense distended abdomen	Endotrache al intubation, resection, anastomosi s	Midgut volvulus (without malrotation) 20 cm proximal to ileocecal valve (the twisted bowel loop became a necrotized cyst mass of 7 cm in diameter)	Death for MOF	Idiopathic
**Steffensen et al. 2008 [2]**	1	15	Case report	Arthrogr yposis	15 w→ ascites<br>25w→ **ascites +dilated bowel**<br>38w→ IUFD	N	↓ FM and IUFD at 38 w	15	Induction for IUFD	VD	38	IUFD due to cardiovascula r failure and shock from midgut volvulus	IUFD	Small bowel volvulus with distal atresia 45 cm from Treitz, large bowel malrotation, no perforation	IUFD	Malrotati on
**Jakhere et al. 2014 [22]**	1	16	Case report	N	**Whirlpool sign, coffee bean sign, bowel dilatation (max 2.3 cm)**	N	N	35	Emergency CS (risk of bowel perforation)	CS	35	ND	Resection, anastomosi s	ileal volvulus with necrosis without malrotation and atresia	U	Idiopathic
**Kornacki et al. 2010 [11]**	1	17	Case report	N	**Hydramnios, Hydrothorax, ascites, dilated bowel** with thick wall, **Anemia**	N	↑PSV MCA (1.73 Mom)	32	Planned CS after corticostero ids	CS	34	Anemia, tense distended abdomen, mild pleural effusion	Blood Transfusion , resection 25 cm of ileum, anastomosi s	Midgut volvulus with significant dilated loop (25 cm) filled with meconium, hypoplastic distal segment of small bowel and colon with adhesion between ileum	Biliar peritonitis at d 17 , sepsis at d 39	Hypoplas tic distal segment of small bowel and colon with adhesion between ileum and liver

														and liver, no malrotation		
**Chung et al. 2013 [23]**	1	18	Case report	Placenta praevia	**Bowel dilatation** (25 mm),**markedly dilated stomach, whirlpool sign, AC >95 th, ascites, peritoneal calcifications**	N	↓ FM (preceding 3 d), ↓CTGv	37	Emergency CS	CS	37	Anemia, tense distended abdomen, tachycardia	Resection, ileostomy	Small bowel volvulus, blood and meconium-stained ascites, no malrotation, no atresia	U	immature intramural ganglion cells in descending and sigmoid colon)
**Noreldeen et al. 2008 [1]**	1	19	Case report	N	**Bowel dilatation** (25 mm), **AC >95 th, ascites, Hydramnios, absence FM**	N	↓ FM, (preceding 2 d), ↓CTGv	31	Emergency CS	CS	31	Anemia, RDS, tense distended abdomen	Blood transfusion, endotracheal intubation, Resection, anastomosis	Volvulus of mild-ileum with 15 cm of necrosis, hemoperitoneum, no malrotation	U	Idiopathic
		20		N	**Whirlpool sign, fluid-meconial level, bowel dilatation, meconium peritonitis**	N	N	27	TOP	VD	28	TOP	TOP	Midgut volvulus, no malrotation	TOP	CF
		21		N	**Whirlpool sign, bowel dilatation, meconium peritonitis**	N	↓ FM	26	ND	VD	37	ND	Resection, anastomosis	Midgut volvulus, atresia, no malrotation	U	Idiopathic
**Bartholomot et al. 2018 [17]**	13	22	Case series	N	**Whirlpool sign, fluid-meconial level, bowel dilatation, meconium peritonitis**	N	↓ FM	34	Emergency CS (non-reassuring FHR)	CS	35	ND	Resection, anastomosis	Midgut volvulus, atresia, no malrotation	Neonatal death	Idiopathic
		23		N	**Whirlpool sign**	N	↓ FM	27	TOP	VD	31	TOP	TOP	Midgut volvulus, no malrotation	TOP	CF
		24		N	**Whirlpool sign, fluid-meconial level, bowel dilatation, meconium peritonitis**	N	↓ FM	28	ND	CS (breach)	38	ND	Resection, anastomosis	Midgut volvulus, atresia, no malrotation	U	Idiopathic

25	Monochorionic diamniotic twin pregnancy	**Bowel dilatation, meconium peritonitis**	N	↓ FM	32	ND	VD	33	ND	Resection, anastomosis	Midgut volvulus, no malrotation	U	Idiopathic mesenteric fusion
26	Monochorionic diamniotic twin pregnancy	**Bowel dilatation, meconium peritonitis**	N	↓ FM	33	ND	VD	33	ND	Spontaneous regression	Midgut volvulus, no malrotation	U	Idiopathic mesenteric fusion
27	N	**fluid-meconial level, bowel dilatation, meconium peritonitis**	N	N	22	ND	VD	35	ND	Resection, anastomosis	Midgut volvulus, atresia, no malrotation	U	Idiopathic
28	Dichorionic diamniotic twin pregnancy	**Whirlpool sign, bowel dilatation,**	N	N	31	ND	CS (breach)	37	ND	Spontaneous regression	Midgut volvulus, no malrotation	Spontaneous regression after birth	Idiopathic
29	N	**Whirlpool sign**	N	↓ FM	31	ND	CS (iterative)	38	ND	Resection, anastomosis	Midgut volvulus, no malrotation	U	Idiopathic
30	N	**Whirlpool sign, bowel dilatation, meconium peritonitis**	N	↓ FM	22	Emergency CS (non-reassuring FHR)	CS	34	ND	Resection, anastomosis	Midgut volvulus, atresia, no malrotation	U	Idiopathic
31	N	**Whirlpool sign, meconium peritonitis**	N	N	27	Induction for ↑ bowel dilatation	VD	36	ND	Resection, anastomosis	Midgut volvulus, atresia, no malrotation	U	Idiopathic
32	N	**Whirlpool sign, fluid-meconial level**	N	↓ FM	28	ND	VD	40	ND	Resection, anastomosis	Midgut volvulus, no malrotation	U	Idiopathic

**Uerpairojkit et al. 2001 [6]**	1	33	Case report	N	**Bowel dilatation arranged in three parallel segments, no peristalsis, whirlpool sign, AC >95 th,**	N	↓ FM, ↓CTGv	33	Preterm labour	VD	33	RDS, Tense distended abdomen	Endotracheal intubation, resection, anastomosis	Gangrenous Ileum volvulus (no atresia, no malrotation), Meckel diverticulum	U	Idiopathic
**Herrera et al. 2020 [24]**	1	34	Case report	Pump twin of TRAP sequence (laser therapy at 18 w)	**Whirlpool sign, bowel dilatation**	N	PPROM at 30 w	26	US FU	CS after PPROM	30	N	Derotation, 10 cm ileum resection, ileostomy	Midgut volvulus with necrosis and perforation, malrotation	U	Malrotation
**Suzumori et al. 1990 [18]**	1	35	Case report	N	**Hydramnios, gross fluid-filled abdominal mass surrounding liver and displacing diaphragm, no peristalsis**	N	Maternal dyspnea for abnormally increased abdomen due to Hydramnios, ↓CTGv	35	FU and paracentesis to identify mass content and decompressing the chest	CS (non-reassuring FHR)	36	RDS, Tense distended abdomen	Endotracheal intubation, resection, anastomosis	Midgut volvulus with necrosis	U	Idiopathic
		36		N	**N**	N	ND	ND	ND	VD	37	N	Resection and double ileostomy	Midgut volvulus with secondary atresia, meconium peritonitis	U	Idiopathic
		37		N	**N**	N	ND	ND	ND	CS (mechanical dystocia)	38	N	Resection and double ileostomy	Midgut volvulus with ileum necrosis	Ileoceal resection at 2 m	Idiopathic
**Raherison et al. 2012 [3]**	10	38	Case series	N	**N**	N	ND	ND	ND	CS (dynamic dystocia)	38	N	Resection-anastomosis	Midgut Volvulus with secondary atresia	U	Primitive Mesenteric breach
		39		N	**N**	N	ND	ND	ND	VD	37	N	Detorsion and laparostomy	Total volvulus with venous ischeemia	U	Narrow mesentere
		40		N	**N**	N	ND	ND	ND	VD	38	N	Resection and double	Midgut volvulus with secondary	U	Idiopathic

										ileostomy	atresia, meconium peritonitis		
41	N	**N**	N	ND	ND	ND	VD	38	N	Derotation and implementation common mesentery	Total volvulus with necrosis	Neonatal death at 7d	Malrotation
42	N	**N**	N	ND	ND	ND	CS (non-reassuring FHR)	31	shock	Medical reanimation evacuation, resection and double ileostomy	Midgut Volvulus with cystic meconial peritonitis	Definitive CR at 13 m	Idiopathic
43	N	**Severe ascites**	ascites without other digestive abnormalities	ND	ND	ND	CS (preeclampsia)	35	RDS	CPAP evacuation, detorsion, resection and double ileostomy	Midgut Volvulus with secondary atresia and cystic meconial peritonitis	Sepsis, cholangitis	Idiopathic
44	N	**Bowel dilatation**	dilatation of multiple jejunal loops with an aspect in of intestinal atresia	ND	ND	ND	VD	38	N	Resection-anastomosis	Midgut Volvulus on atresia with perforation	U	Intestinal atresia
45	N	**Bowel dilatation, gastric dilatation, esophagus dilatation**	stomach dilatation, the duodenal frame as well as the first handle	ND	ND	ND	CS (non-reassuring FHR)	35	N	Resection-anastomosis	Midgut Volvulus on atresia	Sepsis	Intestinal atresia

						jejunale, with presence of meconium in the rectum.										
**Molvarec et al. 2007 [25]**	2	46	Case series	N	**32w→ Bowel dilatation** (max 15 mm) **Hydramnios 36w→ ↑ bowel dilatation, ↑ hydramnios, ascites, AC >95 th**	N	N	32	US FU	Emergency CS (↑ dilatatio, ↑hydramnios, appearance ascites)	36	Tense distended abdomen,	resection-jejunoileostomy)	Jejunoileal volvulus with malrotation, 80 cm necrosis, perforation,meconial peritonitis	SBS	Malrotation
		47		N	**Fluid-filled Bowel dilatation (max 20 mm) → ↑ bowel dilatation, AC >95 th, oligohydramnios, ↑peristalsis)**	N	N	36	Daily FU (NST and US)	CS (↑ bowel dilatation , AC >95 th, oligohydramnios, ↑peristalsis)	36	Tense distended abdomen,	Resection-ileostomy	Midgut volvulus with malrotation and 20 cm of ileum necrosis	U	Malrotation
**Yu et al. 2012 [13]**	1	48	Case report	N	**Bowel dilatation** (max 20 mm)**, whirlpool sign, mild ascites, no peristalsis, mild Hydramnios, 8.1x 5.3x4.6 oval cystic abdominal mass** (PSV not performed)	N	N	37	Delivery	ND	37	Tense distended abdomen, Cullen's sign Anemia	Derotation, resection-anastomosis	Small bowel volvulus with necrosis, markedly proximal distension and distal atresia, hemorraggic ascites, no malrotation	SBS	Idhiopatic /intestinal atresia
**Chouikh et al. 2016 [26]**	2	49	Case series	N	**Large bowel dilatation with deceleration on CTG and loss of peristalsis and vascularization 3 d after**	N	non-reassuring FHR	34	Emergent delivery	CS	34	Tense distended abdomen	Resection, ileal anastomosis+ileostomy for perforation 3w after	Ileum volvulus with necrosis, thick and adhesive meconium	Perforation 3w after surgery	CF
		50				N	N	29		CS	30		Derotation, resection-	Midgut volvulus with secondary	Ileal perforation	CF

				Dichorionic diamniotic twin pregnancy with IUGR of the second twin	**29 w→Bowel dilatation 30 w→ ↑ bowel dilatation, whirlpool sign**				US FU and Emergency CS	(↑ bowel dilatation +whirpool sign)		Tense distended abdomen	ileojejuneal anastomosis	atresia (atresia at the proximal end of twisted volvulus), thick and adhesive meconium	2w after surgery	
**Yip et al. 2017 [27]**	1	51	Case report	N	**21 w→ bowel dilatation (max 6.1 mm) 23 w→ ↑ bowel dilatation (max 8.2mm) 27 w→ ↑ bowel dilatation (max 13 mm) 31w→ ↑ bowel dilatation (max 28 mm), no peristalsis, mild ascites 31 w(+2d)→ bowel dilatation , no peristalsis, ↑ ascites, whirlpool sign, Hydramnios**	N	non-reassuring FHR, ↓ FM at 31 w	21	US FU	Emergency CS (non-reassuring FHR, ↓ FM, worsening US findings)	31	ND	Resection, small bowel anastomosis	Jejuneum volvulus with necrosis and cystic meconium peritonitis, no malrotation	U	Idhiopatic
**Ohuoba et al. 2013 [28]**	1	52	Case report	Mega-cisterna magna	**36 w→ tubular bowel dilatation (coffee bean sign, max 26 mm)**	N	↓ FM (preceding 2 d), ↓CTGv and deceleration at 38 w	36	US FU (twice a w)	Emergency CS (non-reassuring FHR)	38	RDS, Tense distended abdomen	Intubation, Resection (volvulus and intussusception), small bowel anastomosis	Jejuneum 240° volvulus adjacent type 3A intestinal atresia, small bowel intussusception on distal end of atresia	U	Atresia intestinal
**Takacs et al. 2014 [29]**	1	53	Case report	N	**30 w→ mild dilated (11 mm) and hyperechoic bowel 33 w→ ↑bowel dilatation, hyperperistalsis, whirlpool**	N	N	30	US FU	Emergency CS (suspicion of perforation and meconium peritonitis)	34	RDS, Tense distended abdomen	Resection (50 cm necrotic ileum), enterostomy	Intestinal volvulus with necrosis, meconium ileus and peritonitis	SBS	CF

					**sign, Hydramnios 34 w →↑bowel dilatation, whirlpool sign, Hydramnios, ascites, hyperechoic spots**										
**Witter et al. 1986 [30]**	1	54	Case report	N	**N**	N	↓CTGv	34	CS	CS (non-reassuring FHR)	34	RDS, Tense distended abdomen, Cullen's sign Anemia	Intubation, paracentesis (to improve lung inflation), blood transfusion, resection (59 cm distal jejunum and 12 cm proximal ileum), anastomosis	Distal Jejunum-proximal ileum volvulus with hemoperitoneum	U	Idiopathic
**Monard et al. 2017 [31]**	1	55	Case report	N	**27 w→ mild bowel dilatation (14 mm), whirlpool sign, thick and hyperechoic intestinal wall 33 w→ appearance peritoneal calcification 36 w→ ↑bowel dilatation (30 mm) with parietal calcifications and meconium pseudocyst**	N	↓ FM, uterine contractions at 27 w	27	US FU	VD (induction for worsening US and ↓CTGv)	37	RDS, Slightly distended abdomen	Resection (meconium pseudocyst, volvulus, very dilatated small bowel) , ileo-ileal anastomosis	Segmental Small bowel volvulus with meconium pseudocyst secondary to perforation of distal ileum and type II small bowel atresia (5cm above ileocecal valve)	U	Idiopathic
**Hasegawa et al. 1996 [32]**	1	56	Case report	N	**19 w→ Hydramnios, multiple cystic spaces in abdomen, AC >95 th**	N	PPROM at 28 w	19	US FU	Emergency CS (worsening US)	28	RDS, distended abdomen	Intubation, 1° Derotation, diverticulectomy, appendicetomy	Serosanguinous ascites, Meckel's diverticulum without omphalomesenteric band,	Death on d7 for SBS which caused volume and electrolyte imbalance	Partial Malrotation+ Obstruction on Meckel

					**28w→ Hydramnios, multiple cystic spaces in abdomen, ascites, ↑ AC >95 th**								2°resection, jejunostomy	malrotation, small bowel volvulus (140 cm dilatation) with necrosis		diverticulum
**Park et al. 2008 [5]**	1	57	Case report	N	**Bowel dilatation with coffee bean sign**	N	Preterm labor	33	Delivery	VD	33	distended abdomen, Cullen's sign	Detorsion, resection, anastomosis	Midgut volvulus with necrosis and perforation of small bowel , hemorraggic ascites, thick meconium of distal ileum, (no malrotation, atresia or mesenteric defect)	U	Meconium ileum (no CF)
**Aurégan et al. 2015 [33]**	1	58	Case report	Joubert syndrome , IUGR	**Dilated and hyperechoic bowel, gastric dilatation, mild ascites**	Gastric dilatation , bowel dilatation with meconial content, whirlpool sign on common mesentere	↓ FM	33	CS	CS	33	RDS	Reanimation, resection, enterostomy	Small bowel volvulus with necrosis (no malrotation, no mesenteric defect, no CF)	Cardiac arrest on d10	Idiopathic
**Ridley et al. 2018 [34]**	1	59	Case report	Dichorionic diamniotic twin pregnancy	**arch-like intestinal dilatation**	N	N	20	US FU	Spontaneous VD	31	distended abdomen	Resection, jejunostomy	Jejunum volvulus with perforation, pneumoperitoneum and inflammatory adhesions between intestinal loops	U	Idiopathic /Vascular lesion (placenta with signs of hypoxia and ischaemia)
**Samuel et al. 1984 [35]**	1	60	Case report	N	**33 w→ mild hydramnios, bowel loops dilatation, abdominal mass of 5x7 cm under the liver**	N	N	33	US FU	CS to prevent perforation and ischemia	34	distended abdomen	Resection, colonstomy and ileostomy	Mild peritonitis with atresia of terminal ileum. Above the atresia volvulus of terminal ileum filled	U	Atresia of terminal ileum

					**34 w→ hydramnios, bowel loops dilatation, abdominal mass , ↑peristalsis**									with thick meconium		
**Valladares et al. 2010 [10]**	1	61	Case report	Dichorionic diamniotic twin pregnancy	**Meconium pseudocyst** (Abdominal mass occupying the entire left hemiabdomen with mixed echogenicity)	72 × 58 mm, heterogeneous, mesenteric mass without necrosis causing significant distortion of the small intestine to the left	Preterm labor	32	US FU	CS (preterm labor and transverse malposition)	33	ND	Resection volvulated necrotic portion, anastomosis, appendectomy (2 relaparotomy for bowel obstruction)	Perforated Ileum volvulus with secondary meconium pseudocyst	Bowel obstruction 3d after surgery, stenosis of the reanastomis is on d51, SBS	Idiopathic
**Kutuk et al. 2014 [36]**	1	62	Case report	N	**20 w→ snail sign 22 w→ normal intestinal loops, no snail sign, dilated stomach, dilated gastroduodenal junction 36w→ severe bowel loops dilatation, severe hydramnios**	N	PPROM, fetal distress at 36 w	20	US FU	CS (PPROM and fetal distress)	36	ND	Resection atretic jejunum, anastomosis	Intestinal volvulus and secondary jejunoileal atresia	Cholestasis, steatorrhea, weight loss	CF
**Yoo et al. 1999 [37]**	2	63	Case series	N	**Bowel dilatation with whirlpool sign, coffee bean sign distal from whirlpool sign** (produced by closed loop obstruction)**, no flow between dilated loops**	N	N	35	CS for necrosis prevention	CS	35	distended abdomen, Cullen's sign	Derotation, resection of 12 cm, Ladd Band division, appendectomy	Midgut volvulus with malrotation and 12 cm of necrosis, Ladd band from cecum to liver, meconium stained ascites	U	Malrotation

		64		N	**30→ Whirlpool sign with anterior cystic expansion, mild Hydramnios**<br><br>**35→ no whirlpool sign, ↑Hydramnios, persistent cystic mass, ↑proximal bowel dilatation**	N	N	30	US FU	VD	37	distended abdomen	Derotation, resection of atretic and stenotic segment, anastomosis, appendectomy, replacement of cecum in left abdomen	Midgut volvulus with distal atresia and mesenteric defect, aneurismatic bowel proximal to atresia, multiple stenosis, malrotation	U	Malrotation
**Finley et al. 1992 [38]**	1	65	Case report	N	**13 w→ umbilical herniation**<br>**15 w→ normal cord insertion**<br>**20w→ small bowel dilation, ↑peristalsis**	N	N	13	US FU	VD	36	Tense and distended abdomen	Resection, anastomosis	Large midgut volvulus with necrosis (only 15 cm of small bowel left)	SBS	Delayed return of midgut to abdomen
**Miyakoshi et al. 2001 [39]**	1	66	Case report	N	**multiple hypoecogenic and mildly dilated loops with strong peristalsis, convoluted mass of enlarged hyperechogenic loops without peristalsis**	Mildly dilated bowel loops were of homogeneous high signal intensity, while a convoluted mass of the enlarged loops was homogeneous with intermediate signal intensity (midgut volvulus with hemorrhagic change)	↓ FM, PPROM at 36w	34	US FU	CS (non-reassuring FHR)	36	Tense and distended abdomen	Resection, ileostomy	Small bowel volvulus with hemorraggic necrosis of jejuneum and distal ileal atresia	U	Ileal atresia

**Jéquier et al. 2003 [9]**	1	67	Case report	Twin pregnancy	**27w →** 5.5 cm complex **abdominal mass , bowel loops dilatation**<br><br>**29 w→ Hydramnios, ↑bowel dilatation , abdominal mass became cystic**	N	N	27	US FU	ND	33	ND	Resection, anastomosis	Midgut volvulus around persistent entero-mesenteric umbilical connection with subsequent bowel necrosis, perforation and cystic peritonitis (no malrotatiom)	U	persistent entero-mesenteric umbilical connection
**Allahdin et al. 2004 [40]**	1	68	Case report	N	**N**	N	IUFD	39	Spontaneous delivery	VD	39	N	N	Ischemic hemorrhagic necrosis secondary to midgut volvulus	IUFD	Idiopathic
**Cloutier et al. 1983 [41]**	1	69	Case report	N	**10 cm cystic abdominal** mass with septations and fluid-debris level, **Hydramnios**	N	PPROM	35	Spontaneous delivery	VD	35	distended abdomen, Cullen's sign	reanimation	Midgut volvulus with necrosis of twisted intestine and dissolution of twisted loops into pseudocyst	Neonatal death on d3	Malrotation
**Schiermeier et al. 2007 [42]**	1	70	Case report	N	**Stomach dilatation**	N	↓ FM, ↓CTGv	32	CS	CS (non-reassuring FHR)	32	ND	Resection (17 cm of jejunum), double barrel jejunostomy	Jejunum volvulus with necrosis	U	Idiopathic
**Has et al. 2002 [8]**	1	71	Case report	N	**34 w→ severe Hydramnios, mild ascites, whirlpool sign (no flow signal) 35w→ disappearance whirlpool sign, ↑bowel dilatation, thick ascites of mixed echogenicity**	N	Preterm labor	34	US FU		35	distended abdomen, Cullen's sign	Resection 30 cm, double-barrel anastomosis	Midgut volvulus with necrosis and perforation, atresia inferior level to the volvulus, duplication of intestine with blind end inside necrotic volvulus resected	Death on d36 (intraventricular hemorrhage , sepsis)	Intestinal duplication/atresia/idiopathic
**De Felice et al. 1997 [43]**	1	72	Case report	N	**Bowel loops dilatation**	N	Preterm labor	27	Spontaneous VD	VD	27	distended abdomen, failure to pass meconium, bilious gastric aspirate	Resection, anastomosis	Midjejuneal volvulus (no malrotation, no mesenteric defects)	U	Idiopathic

**Neis et al. 1983 [44]**	1	73	Case report	N	**Hydramnios**	N	Preterm labor	33	Spontaneous VD	VD	33	RDS, distended abdomen	Reanimation	Volvulus with complete necrosis of ileum with subsequent intrauterine development of a pseudocyst, ascites	Death on d1	Mesenteric defect/idiopathic
**Taba et al. 2010 [45]**	1	74	Case report	N	**Severe meconium ascites, giant pseudocyst,**	Definitive diagnosis of meconium peritonitis (distinction meconium from peritoneal fluid)	↓ FM (preceding 24h), Non-reassuring CTG	35	Emergency CS (non-reassuring FHR)	CS	35	RDS, distended abdomen, Cullen’s sign	Intubation, paracentesis, resection (46 cm), anastomosis	Small bowel volvulus with 46 cm necrosis, meconium peritonitis, (no malrotation)	Cholestasis	Idiopathic
**Ogunyemi et al. 2000 [46]**	1	75	Case report	Dichorionic diamniotic twin pregnancy	**Hydramnios, dilated stomach, bowel double loops dilatation with central obstruction, multiple bowel loops dilatation (17-28 mm)**	N	↓CTGv	36	Emergency CS (non-reassuring FHR	CS	36	Bilious stained amniotic fluid	25 cm ileum resection , ileostomy	Ileojejuneal volvulus, ileal atresia with necrotic ileum, peritonitis with hemorrhagic fluid (No malrotation, no mesenteric defect)	SBS	Ileal atresia
**Lee et al. 2011 [47]**	1	76	Case report	N	**21w → abdominal anechoic cyst** 20x19 mm **32w→ ↑cyst size** 27x19mm **34w→↑cyst size** 30x26mm **38w→ cyst became with homogeneous moderate echogenicity, bowel loops dilatation**	large oval cyst in the lower abdomen with internal low signal intensity debris on T2-weighted images and diffusely dilated small bowel loops	N	21	US FU	CS	38	RDS, distended abdomen	adhesiolysis, cyst dissection, meconium aspiration, Derotation, ileostomy, no resection	Midgut volvulus, Meconium cyst with distal ileal atresia (type II), proximal small bowel perforation	U	Ileal atresia (type II)

**Leung et al. 2001 [19]**	1	77	Case report	Dichorionic diamniotic twin pregnancy	**33w→ ascites with intraperitoneal debris (meconium peritonitis), AC >95 th 34 w→ ascites, ↑AC (40 cm), Hydramnios, no stomach visible, diaphragm elevation**	N	↓ FM, non-reassuring CTG	33	US and NST FU, paracentesis (to prevent dystocia)+ amnioreduction at 34w	Spontaneous VD 10 h after intervention	34	RDS, distended abdomen	Intubation, 5cm distal ileum resection, ileostomy	Distal ileal volvulus with secondary perforation, meconium peritonitis	Stomal obstruction	Idiopathic
**Alvarez et al. 1991 [48]**	1	78	Case report	twin pregnancy, IUGR	**30w→ cystic abdominal mass, oligohydramnios 34w→ ↑cyst size (71x73 mm), diaphragm elevation**	N	N	30	Weekly US and NST FU	CS (worsening US)	34	distended abdomen, failure to pass meconium, bilious gastric aspirate	Resection, anastomoisis	perforated congenital volvulus of the ileum and secondary cystic meconium peritonitis.(no malrotation, no atresia)	SBS	Idiopathic
**Baxi et al. 1983 [49]**	1	79	Case report	IUGR	**34 w→ hydramnios, mild ascites, cystic mass in right hypocondrium, ↑AC 34w+3d→ ↑AC,↑ hydramnios, marked ascites, diaphragm elevation, no more cystic mass (probably for bowel perforation)**	N	N	34	US FU, induction of labor for worsening US (amnioreduction+paracentesis to facilitate VD and ↓fetal distress)	VD	35	distended abdomen	Resection, anastomosis	Meconium ileum with secondary intrauterine small bowel volvulus, necrosis,perforation, pseudocyst formation and meconium peritonitis, microcolon	U	Microcolon
**Casaccia et al. 2003 [50]**	2	80	Case series	ND	**Bowel dilatation**	N	ND	25	ND	ND	ND	ND	ND	Small bowel volvulus	ND	Idiopathic
		81		ND	**Bowel dilatation**	N	ND	27	ND	ND	ND	ND	ND	Small bowel volvulus	ND	Idiopathic

**Chen et al. 2022 [51]**	1	82	Case report	High torsion of umbilical cord	30 w →mixed echogenic **abdominal mass** of 73 mm × 52 mm, **Hydramnios** 31 w→ ↑abdominal mass to 82 mm × 53 mm, **Hydramnios** 32 w→ ↑↑**abdominal mass, fluid-filled loop of bowel**, and **bowel dilatation** of the adjacent loops, **Hydramnios**	Fetal **intra-abdominal mass** with a **line-like separation** and **abnormal nodular signals**. Suspicion of **cystadenoma.**	PPROM, ↓CTGv	30	US FU	Emergency CS (non-reassuring FHR)	32	Tense distended abdomen	resection, anastomosis	Midgut volvulus with necrosis	U	Idiopathic
**Jain et al. 2022 [52]**	1	83	Case report	N	**Hydramnios, Whirlpool sign, bowel dilatation (23 mm)**	N	↓ FM	33	Emergency CS	CS	33	ND	Untwisting of the volvulus followed by Ladd's procedure	Midgut volvulus with malrotation (no ischemia, no necrosis)	U	Malrotation
**Matsushima et al. 2022 [53]**	1	84	Case report	N	34w→ **bowel dilatation (24mm)** 37 w→ worsening **bowel dilatation**	N	↓ FM, ↓CTGv and CTG decelarations	34	US FU	Emergency CS (non-reassuring FHR)	37	Bilious drainage from stomach, distended abdomen, bluish/white skin discolouration of the abdominal wall, RDS, abdominal compartment syndrome	Endotracheal intubation, resection, anastomosis between the jejunum and ascending colon	Midgut volvulus with extensive necrosis, hemorrhagic ascites (no malformation)	U	Idiopathic
**Li et al. 2022 [54]**	12	85	Case series	N	**Bowel dilatation, Hydramnios, meconium peritonitis, Fluid-filled levels, ascites, ↑PSV MCA**	N	↑PSV MCA	35	CS	CS	35	preoperative hemoglobin 92 g/L	ND	Terminal ileum volvulus with 35 cm of necrosis	SBS	Idiopathic
		86		N	**Bowel dilatation,**	N	ND	37	CS	CS	37	ND	ND	Terminal ileum volvulus with	SBS	Mesentery dysplasia

		**Whirpool sign, coffe-bean sign, Hydramnios, meconium peritonitis, Fluid-filled levels, ascites**									35 cm of necrosis		
87	N	**Bowel dilatation, Whirpool sign, meconium peritonitis, ascites**	N	ND	28	US-FU	CS	32	ND	ND	Ileal volvolus with necrosis	U	Malrotati on
88	N	**Bowel dilatation, Hydramnios, Coffe bean sign, meconium peritonitis, Fluid-filled levels, ascites, ↑PSV MCA**	N	↑PSV MCA	34	CS	CS	34	preoperative hemoglobin 103 g/L	ND	Ileaum volvolus with 35 cm of necrosis	U	Mesenter y dysplasia
89	N	**Bowel dilatation, Hydramnios, whirpool sign, meconium peritonitis, ascites, ↑PSV MCA**	N	↑PSV MCA	36	CS	CS	36	preoperative hemoglobin 98 g/L	ND	Ileaum volvolus with 20 cm of necrosis	U	Malrotati on
90	N	**Bowel dilatation, ascites , meconium peritonites**	N	ND	30	US-FU	CS	34	ND	Resection of 40 cm of midgut	Ileum volvulus with necrosis	SBS	Ileal atresia
91	N	**Bowel dilatation, Hydramnios, ascites , meconium peritonites**	N	ND	35	US-FU	VD	37	ND	35 cm of intestine resected	Jejunum volvulus with necrosis	U	Mesenter y dysplasia

92	N	**27w→ Bowel dilatation, Hydramnios, whirpool sign, meconium peritonitis and ascites 29w→ appearance Coffee bean sign**	N	ND	27	US-FU	CS	34	ND	ND	Ileum volvulus with necrosis	U	Malrotation
93	N	**Bowel dilatation, meconium peritonitis and ascites**	N	ND	28	US-FU	CS	34	ND	ND	Jejunum volvulus with necrosis	U	Malrotation
94	N	**Bowel dilatation, Hydramnios, meconium peritonitis, ascites, ↑PSV MCA**	N	↑PSV MCA	22	US-FU	CS	34	ND	ND	Ileum volvulus with necrosis	U	Ileum atresia
95	Biliary atresia type III	**Bowel dilatation, Coffe bean sign, abdominl cystic mass, Hydramnios, meconium peritonitis, ascites, ↑PSV MCA**	N	↑PSV MCA	32	US-FU	CS	35	preoperative hemoglobin 95 g/L	ND	Jejunum volvulus with necrosis of 30 cm; the intestine was broken into two segments; large ascites and severe adhesions of intestine were present.	Intestinal obstruction 3 w after operation	Jejunum atresia
96	N	**Bowel dilatation, whirpool sign, meconium peritonitis, fluid-filled levels and ascites**	N	ND	21	US-FU	CS	33	ND	ND	Ileum volvulus with necrosis	U	Malrotation

		97		Ventricular septal defect, hexadactyly, Trisomy 21	**Whirlpool sign, bowel Dilatation**	N	ND	26	TOP	VD	27	ND	TOP	Midgut volvulus	TOP	Malrotation
		98		Renal duplication	**Whirlpool sign, bowel Dilatation intestinal echo enhancement**	N	ND	27	TOP	VD	27	ND	TOP	Midgut volvulus	TOP	Malrotation
		99		N	**Whirlpool sign, bowel Dilatation, ascites intestinal echo enhancement**	N	ND	27	TOP	VD	28	ND	TOP	Midgut volvulus	TOP	Malrotation
**Yang et al 2022 [55]**	11	100	Case series	N	**Whirlpool sign, bowel Dilatation intestinal echo enhancement**	N	ND	30	US-FU	VD	39	ND	Ladd's surgery	Midgut volvulus	U	Malrotation
		101		N	**Whirlpool sign, bowel Dilatation intestinal echo enhancement**	N	ND	30	US-FU	VD	39	ND	Ladd's surgery	Midgut volvulus	U	Malrotation
		102		N	**Whirlpool sign, bowel Dilatation intestinal echo enhancement**	N	ND	31	US-FU	VD	39	ND	Ladd's surgery	Midgut volvulus	U	Malrotation
		103		N	**Whirlpool sign, bowel Dilatation, intestinal echo enhancement**	N	ND	32	US-FU	VD	38	ND	Ladd's surgery	Midgut volvulus	U	Malrotation

104	N	**Whirlpool sign, ascites, bowel Dilatation, intestinal echo enhancement**	N	ND	33	CS	CS	33	Abdominal cyanosis, edema, and respiratory instability	Ladd's surgery, resection and anastomosis for atresia	Midgut volvulus complicated with ileal atresia	SBS	Malrotation
105	N	**Whirlpool sign, bowel dilatation, ascites**	N	ND	33	CS	CS	34	Abdominal cyanosis, edema, and respiratory instability	Ladd's surgery, resection and anastomosis for atresia	Midgut volvulus complicated with ileal atresia	SBS	Malrotation
106	N	**Whirlpool sign, ascites, bowel Dilatation, intestinal echo enhancement**	N	ND	34	CS	CS	34	Abdominal cyanosis, edema, and respiratory instability	Ladd's surgery, resection and anastomosis for atresia	Midgut volvulus complicated with ileal atresia	SBS	Malrotation
107	N	**Whirlpool sign, bowel dilatation, intestinal echo enhancement**	N	ND	35	US-FU	VD	38	ND	Ladd's surgery	Midgut volvulus	U	Malrotation

GA, gestational age; W, weeks; Y, years; D, days; M, months; H, hours; FU, follow up; N°, case number; CS, cesarean section; VD, vaginal delivery; MRI, magnetic resonance imaging; US, ultrasound; ND, no data available; N, none; FM, fetal movements; CTGv, cardiotocography variability; RDS, respiratory distress syndrome; CTG, cardiotocography; NICU, neonatal intensive care unit; CPAP, continuous positive airway pressure; LPT, laparotomy; CF, cystic fibrosis; FHR, fetal heart rate; PSV, peak systolic velocity; MCA, middle cerebral artery; IUFD, intrauterine fetal death; AC, abdominal circumference; TOP, termination of pregnancy; TRAP, twin reversed arterial perfusion; NST, non-stress tests; IUGR, intrauterine grow restriction; PPROM, preterm premature rupture of membranes; U, uneventful; SBS, short bowel syndrome; MOF. multiorgan failure; CR, continuity restoration.
