# Supplementary material for: Antenatal Diagnosis and Management of Fetal Intestinal Volvulus: Case Series and Literature Review
Source: J Clin Med. 2023 Jul 20;12(14):4790. doi: 10.3390/jcm12144790 (PMC10381374; doi:10.3390/jcm12144790)
Supplement: Supplementary file 1 [file jcm-12-04790-s001.zip › Table S3.pdf]

Table S3. Ultrasounds features of fetal intestinal volvulus (Review of Literature)

	Bowel dilatation	Whirlpool sign	Ascites	Hydramnios	Meconium Pseudocyst	Cystic abdominal mass with septations	Coffee bean sign	AC >95 th	Diaphragm elevation	Peritoneal calcifications	Stomach dilatation	Fluid-meconium level	Hydrops	
1	+		+											MULTIPLE US FINDINGS
2	+		+											
3	+		+											
4	+			+										
5	+			+										
6	+			+										
7	+			+										
8	+				+									
9	+										+			
10	+	+	+											
11	+	+	+											
12	+	+	+											
13	+	+	+	+										
14	+	+		+		+								
15	+	+	+	+		+								
16	+	+	+	+						+				
17	+	+	+	+						+				
18	+	+												
19	+	+			+					+				
20	+			+		+						+		
21	+			+		+						+		
22	+			+	+									
23	+		+					+						
24	+		+	+				+						
25	+		+							+				
26	+		+							+				
27	+	+					+							
28	+	+						+			+			
29	+	+												
30	+	+	+							+		+		
31	+	+	+							+		+		
32	+	+	+							+		+		
33	+	+	+							+				
34	+	+	+							+				
35	+	+	+							+				
36	+						+	+			+			
37	+				+		+							
38	+			+									+	
39				+		+						+		
40				+		+						+		
41	+		+	+				+						
42	+							+				+		
43		+	+					+		+	+			
44		+										+		
45		+	+		+					+				
46	+		+	+				+						
47			+	+	+			+	+					
48					+				+					
49			+	+				+	+					
50			+		+									
51	+	+												
52	+			+		+						+		
53	+			+										
54	+		+	+								+		
55	+	+	+	+			+					+		
56	+	+	+											
57	+		+	+			+					+		
58	+	+	+	+										
59	+		+											
60	+		+	+										
61	+	+	+	+			+							
62	+		+											
63	+		+	+										
64	+		+	+		+	+							
65	+	+	+									+		
66	+	+												
67	+	+												
68	+	+	+											
69	+	+												
70	+	+												
71	+	+												
72	+	+												
73	+	+	+											
74	+	+	+											
75	+	+	+											
76	+	+	+											
77	+													SINGLE US FINDINGS
78	+													
79	+													
80	+													
81	+													
82	+													
83	+													
84	+													
85	+													
86	+													
87	+													
88		+												
89		+												
90			+											
91				+										
92					+									
93						+								
94							+							
95							+							
96											+			
97											+			
98														NO US FINDINGS
99														
100														
101														
102														
103														
104														
105														
106														
107														
Tot.	78	41	45	31	9	9	9	10	3	13	6	14	1	

US, ultrasound; AC, abdominal circumference; Tot, total number of cases.
